# Supplementary material for: Adolescent COVID-19 Vaccine Decision-Making among Parents in Southern California
Source: Int J Environ Res Public Health. 2022 Apr 1;19(7):4212. doi: 10.3390/ijerph19074212 (PMC8998887; doi:10.3390/ijerph19074212)
Supplement: Supplementary file 1 [file ijerph-19-04212-s001.zip › Adolescent Focus Group Discussion Guide.pdf]

Child Decision-Making About COVID19 Vaccine
Discussion Guide [English Version]

INTRODUCTION

We are talking with parents and their middle and high-school aged children to learn more about the parent-adolescent COVID19 vaccine decision making process. Today, we are interested in learning more about your perspective on the COVID-19 vaccines and vaccines in general.

We'd like to hear your thoughts to better understand how your family makes vaccine decisions and where you turn to for COVID-19 vaccine information. We would like to video record the focus group discussion for accuracy purposes and so that we can review the discussion in more detail after the focus group. The information you share with us today will remain confidential. That means, if we report something in report then will report it in such a way to only talk about generalizations and not mention any single individuals' comments. We are most interested in hearing and learning about your thoughts and experiences. Please remember that everything you tell us is confidential.

OK…let's get started.

WARM UP / ICE BREAKER

- Introduce yourself and tell us your grade.
- Tell us about your current school situation.

PART I: CHILDREN and VACCINES in GENERAL

1) Talk to me about the last time you received a shot.
    - Which shot was it? For example the flu shot or another shot that is recommended for middle and high schoolers (i.e. Tdap, HPV, meningitis)
    - Do you remember getting that shot before school started?
    - What was that like? (i.e. any fear of needles)

PART II: CHILDREN and the COVID-19 VACCINE

2) Talk to me about the COVID-19 vaccine.
    - What are your thoughts about it?
    - What have you heard about it?
    - Who have you talked to about the vaccine? For example parents, friends, other family members, teachers, etc.
    - What have you talked about with this person?
    - What do you think about what they said?
    - When the vaccine is offered to you, do you think you'd want to take it?

PART III: CHILDREN and THEIR QUESTIONS

3) If you have any questions about the COVID-19 vaccine, what would you want answers about?

Part IV: CHILDREN and SEEKING INFORMATION

4) Tell me about who you would turn to find out more about the COVID-19 vaccine.

- For example, teachers, friends, family, Internet, social media
