# Supplementary material for: Adolescent COVID-19 Vaccine Decision-Making among Parents in Southern California
Source: Int J Environ Res Public Health. 2022 Apr 1;19(7):4212. doi: 10.3390/ijerph19074212 (PMC8998887; doi:10.3390/ijerph19074212)
Supplement: Supplementary file 1 [file ijerph-19-04212-s001.zip › Adolescent Survey.pdf]

<u>**Survey - Adolescent**</u>

Targeted communication about vaccinating

1) At least one of my parents has talked to me about vaccines in general.
   - Strongly agree
   - Agree
   - Neither agree or disagree
   - Disagree
   - Strongly disagree

2) At least one of my parents has talked with me about the COVID-19 vaccine.
   - Strongly agree
   - Agree
   - Neither agree or disagree
   - Disagree
   - Strongly disagree

3) At least one of my parents has shown me information on the Internet, TV or in the news about the COVID-19 vaccine.
   - Strongly agree
   - Agree
   - Neither agree or disagree
   - Disagree
   - Strongly disagree

4) At least one of my parents has asked me about my thoughts and opinions about the COVID-19 vaccine.
   - Strongly agree
   - Agree
   - Neither agree or disagree
   - Disagree
   - Strongly disagree

General parent-child communication openness

5) At least one of my parent(s) listens to my point of view.
   - Strongly agree
   - Agree
   - Neither agree or disagree
   - Disagree
   - Strongly disagree

6) At least one of my parent(s) says it’s important to get my ideas across even if others don’t like it.

- Strongly agree
- Agree
- Neither agree or disagree
- Disagree
- Strongly disagree

7) At least one of my parent(s) asks for my opinion when our family is deciding something.
    - Strongly agree
    - Agree
    - Neither agree or disagree
    - Disagree
    - Strongly disagree

Frequency of communication about vaccines

8) How often in the past year did you talk to your parent(s) about the COVID-19 vaccine?
    - Never
    - Rarely
    - Sometimes
    - Often
    - Very often

9) How often in the past year did you talk with your parent(s) about other vaccines you may need? For example, the flu, meningococcal (meningitis), or HPV vaccine.
    - Never
    - Rarely
    - Sometimes
    - Often
    - Very often

Parental style

10) In general, how are the most important health decisions made between you and parent(s)?
    - No parent involvement at home
    - They tell me exactly what to do
    - They ask my opinion, but they have the final say
    - They discuss the decision with them, but then let me decide
    - They trust them to decide for myself
    - They don’t care what I do, so I decide for myself

11) When it comes to the COVID-19 vaccine, my parent will include me in the decision-making process.
    - Strongly agree
    - Agree

- Neither agree or disagree
- Disagree
- Strongly disagree

COVID vaccine intent

12) I want to get vaccinated against COVID-19.
    - Strongly agree
    - Agree
    - Neither agree or disagree
    - Disagree
    - Strongly disagree

Positive Expectancies

13) I think the COVID-19 vaccine will protect me from the COVID-19 virus.
    - Strongly agree
    - Agree
    - Neither agree or disagree
    - Disagree
    - Strongly disagree

14) I think the COVID-19 vaccine will stop me from passing the COVID-19 virus to others.
    - Strongly agree
    - Agree
    - Neither agree or disagree
    - Disagree
    - Strongly disagree

Vaccinating under conditions of Uncertainty

15) I think the COVID-19 vaccine is safe
    - Strongly agree
    - Agree
    - I don't know
    - Disagree
    - Strongly disagree

Information sources

16) Indicate how much you trust vaccine information from family. For example, your parents or other relatives.
    - Not at all

- Slightly
- Somewhat
- Very much
- Extremely

17) Indicate how much you trust vaccine information from medical professionals. For example, doctors.
    - Not at all
    - Slightly
    - Somewhat
    - Very much
    - Extremely

18) Indicate how much you trust vaccine information from new media websites. For example, local or national news.
    - Not at all
    - Slightly
    - Somewhat
    - Very much
    - Extremely

19) Indicate how much you trust vaccine information from social media. For examples, social networking sites and online blogs or vlogs.
    - Not at all
    - Slightly
    - Somewhat
    - Very much
    - Extremely

20) Indicate how much you trust vaccine information from friends.
    - Not at all
    - Slightly
    - Somewhat
    - Very much
    - Extremely

21) Indicate how much you trust vaccine information from public health websites. For example, websites from the CDC, World Health Organization (WHO), or Orange County Department of Health
    - Not at all
    - Slightly
    - Somewhat
    - Very much
    - Extremely

Ranking of vaccine concerns

22) When thinking about **vaccinating yourself**, please rank your top five COVID-19 vaccine **concerns**

1. Lack of support by important others (Example: parents, family, or friends)
2. Concerns over potential short term side effects or reactions (Example: pain or swelling at the injection site, headache)
3. Concerns over potential long term side effects
4. Concerns over the ingredients that make up the vaccine
5. Concerns over vaccine safety
6. Concerns over how well the vaccine works
7. Trust in how the vaccine was developed
8. Fear of receiving too many vaccines
9. Personal medical history (Example: a health condition)
10. Personal allergies
11. Preference for natural immunity
12. Low perceived risk of getting COVID-19
13. COVID-19 isn't a very severe disease for kids
14. COVID-19 isn't a very severe disease for adults
15. I've already had COVID-19
16. Cost
17. Fear of needles
18. Uncertainty about vaccine benefits
19. Not enough vaccine information or a lack of access to vaccine information
20. Social media or Internet stories or comments
21. Other (please indicate) ____

Ranking of vaccine motivators

23) When thinking about **vaccinating yourself**, please rank the top five things that would **motivate** you to get the COVID-19 vaccine

1. Confidence in vaccine safety
2. Confidence in how well the vaccine works
3. Confidence in how the vaccine was developed
4. Important others think it's a good idea to vaccinate (Example: parents, family, or friends)
5. A healthcare provider tells me it's a good idea to vaccinate
6. Personal medical history (Example: High risk health condition)
7. Personal allergies
8. Not worried about short term side effects
9. Not worried about potential long term side effects
10. Not worried about the ingredients that make up the vaccine
11. I think I may be at high of risk of getting COVID-19
12. I think that COVID-19 can really make me sick

13. No cost
14. Easy access
15. Social benefit (Example: things going back to normal)
16. It’s important to protect others (Example: herd immunity, protecting those you live with)
17. Certain about vaccine benefits
18. Having enough information or access to information
19. Social media or Internet
20. Other (please indicate below) ____
