# Supplementary material for: Adolescent COVID-19 Vaccine Decision-Making among Parents in Southern California
Source: Int J Environ Res Public Health. 2022 Apr 1;19(7):4212. doi: 10.3390/ijerph19074212 (PMC8998887; doi:10.3390/ijerph19074212)
Supplement: Supplementary file 1 [file ijerph-19-04212-s001.zip › Parent Focus Group Discussion Guide.pdf]

Parent Decision-Making About COVID19 Vaccine
Discussion Guide [English Version]

INTRODUCTION

We are talking with parents and their middle and high-school aged children to learn more about the parent-adolescent COVID19 vaccine decision making process. Today, we are interested in learning more about your perspective on the COVID-19 vaccines and vaccines in general.

We'd like to hear your thoughts to better understand how your family makes vaccine decisions and where you turn to for COVID-19 vaccine information. We would like to video record the focus group discussion for accuracy purposes and so that we can review the discussion in more detail after the focus group. The information you share with us today will remain confidential. That means, if we report something in report then will report it in such a way to only talk about generalizations and not mention any single individuals' comments. We are most interested in hearing and learning about your thoughts and experiences. Please remember that everything you tell us is confidential.

OK…let's get started.

WARM UP / ICE BREAKER

- Introduce yourself, your name and your children – are they in middle and or high school.

- Tell us about your child's current school situation.

PART I: PARENT DECISION MAKING and PARENT COMMUNICATION WITH ADOLESCENT CHILDREN ABOUT VACCINATING in GENERAL

1) Parents make many decisions about whether or not to vaccinate. Tell me about how you made the decision about whether or not to vaccinate your child the last time you were asked to do so? (eg, flu vaccine the past year, 3 adolescent vaccines recommended for middle school entry Tdap, HPV, meningitis)

    - Walk me through how you decided to vaccinate/not vaccinate your child/children last time, who you talked to, what you thought about what they said, did any events or conversations result in you getting your children vaccinated?

    - What prompted your decision to get your child/children vaccinated/not vaccinated? (e.g., physician recommendation, school requirement, sport or event requirement, experiences with allergic reaction, annual event/ritual)

    - Who did you turn to for information? (e.g., doctor, spouse, child)

    - How did those conversations go?

    - What did you think about those conversations, what they said?

    - Tell me about the things you consider (e.g., health of your child, opinion of important others, preference for natural immunity, school requirements)

    - What experiences or events contribute to your decision-making process? (e.g., school requirements, sport requirements, experiences of children having reactions)

    - Tell me about which vaccines you decided your child should take and which ones they should not. Why is that? (vaccine specific hesitancy)

PART II: PARENT PERSPECTIVES/DECISION-MAKING ABOUT COVID19 VACCINE

2) Please share with us your thoughts about the COVID19 vaccine. Describe to me any informal or formal conversations you have had so far about the COVID19 vaccines?

    - Have you talked about COVID vaccine with a healthcare professional?
    - What have important others said?
    - What did you think about what they said?
    - Have you discussed or read about vaccine information online? What sources have you used (Facebook, Twitter, Instagram, email, family group chats etc.)

3) Describe to me what you know about safety and effectiveness in adults and children *[Confidence]*

    - Talk about any concerns you may have (e.g. safety, effectiveness, duration of protection, mistrust of government or pharmaceutical companies, policies that enforce vaccination). *[Vaccine confidence/Trust]*
    - If you were to rank your concerns…..

4) Describe your perspective on whether your child needs the COVID19 vaccine and whether you think your child is at risk for getting COVID19. *[Complacency]*

5) Tell me how confident you feel that you will be able to get your children vaccinated once it becomes available? Why is that? *[Constraints]*

    - Tell me about any potential barriers that might impact you (e.g. insurance, cost, finding out where and when you can access it, availability or shortages, etc.) *[Constraints]*

6) Tell me how helpful the vaccine will be for you, your child, your household or your community (e.g. herd immunity) *[Collective responsibility; benefits of vaccine]*

7) Explain to me what you plan to do about vaccinating your child once it becomes available and recommended? *[Calculation; risk/benefit weighing]*

- Tell me about the things that you consider in your decision (e.g. the health of your child, the opinions of others, preference for natural immunity, etc.) *[complacency: perceived risk] [calculation: information seeking, weighing risk/benefit] [uncertainty]*

8) Who would be the person whose recommendation to take the vaccine or not would have the most impact on your decision?

PART III: VACCINE ACCEPTABILITY

9) What information or actions if any, would make getting the vaccine more acceptable?

    - For example, more information, a recommendation, rigorous testing, testing in children, a vaccine campaign, etc.

PART IV: COVID-19 VACCINE INFORMATION SOURCES

10) Where or who do you turn to for information about the COVID19 vaccine?

    - For example, traditional media, social media, the internet, a healthcare professional, family, friends, etc.

PART V: YOUNGER CHILDREN (IF APPLICABLE)

11) For those of you who have children under the age of 12 in the home, how are your decisions about whether or not to have them take the vaccinate the same or different?

    - What additional information might you need to make that decision?

12) Would you be open to your child under 12 taking part in a study on the vaccine?

    - What would be some deciding factors for your decision (eg, worries about safety, not trusting research, wanting my child to get vaccinated early)?

DEBRIEF

13) Is there anything else you would like to share that we haven’t asked you?
