# Supplementary material for: Adolescent COVID-19 Vaccine Decision-Making among Parents in Southern California
Source: Int J Environ Res Public Health. 2022 Apr 1;19(7):4212. doi: 10.3390/ijerph19074212 (PMC8998887; doi:10.3390/ijerph19074212)
Supplement: Supplementary file 1 [file ijerph-19-04212-s001.zip › Parent Survey.pdf]

Survey - Parent

Targeted communication about vaccinating

1) I have talked with my adolescent child about vaccines in general. For example, the flu, meningococcal (meningitis), or HPV vaccine.
    - Strongly agree
    - Agree
    - Neither agree or disagree
    - Disagree
    - Strongly disagree

2) I have talked with my adolescent child about the COVID-19 vaccine.
    - Strongly agree
    - Agree
    - Neither agree or disagree
    - Disagree
    - Strongly disagree

3) I have shown my adolescent child information on the Internet, TV or news about the COVID-19 vaccine
    - Strongly agree
    - Agree
    - Neither agree or disagree
    - Disagree
    - Strongly disagree

4) I have asked my adolescent child about their thoughts and opinions regarding the COVID-19 vaccine.
    - Strongly agree
    - Agree
    - Neither agree or disagree
    - Disagree
    - Strongly disagree

General parent-child communication openness

5) I listen to my adolescent child's point of view.
    - Strongly agree
    - Agree
    - Neither agree or disagree
    - Disagree
    - Strongly disagree

6) I tell my adolescent child that it's important to get their ideas across even if others don't like it.

- Strongly agree
- Agree
- Neither agree or disagree
- Disagree
- Strongly disagree

7) I ask my adolescent child for their opinion when our family is discussing something.
    - Strongly agree
    - Agree
    - Neither agree or disagree
    - Disagree
    - Strongly disagree

Frequency of communication about vaccines

8) How often in the past year did you communicate with your adolescent child about the COVID-19 vaccine?
    - Never
    - Rarely
    - Sometimes
    - Often
    - Very often

9) How often in the past year did you communicate with your adolescent child about other vaccines they may need? For example, the flu, meningococcal (meningitis), or HPV vaccine.
    - Never
    - Rarely
    - Sometimes
    - Often
    - Very often

Parental style

10) In general, how are the most important health decisions made between you and your adolescent child?
    - I am not involved at home
    - I tell them exactly what to do
    - I ask for their opinion, but I have the final say
    - I discuss the decisions with them, but then let them decide
    - I trust them to decide for themselves
    - I don’t care what they do, so I let them decide for themselves

11) When it comes to the COVID-19 vaccine, I will include my adolescent child in the decision-making process.
    - Strongly agree
    - Agree
    - Neither agree or disagree
    - Disagree
    - Strongly disagree

COVID vaccine intent

12) Do you plan on vaccinating your child against COVID-19?
    - Yes
    - No
    - I'm not sure yet

Positive Expectancies

13) I think the COVID-19 vaccine will protect my adolescent child from the COVID-19 virus.
    - Strongly agree
    - Agree
    - Neither agree or disagree
    - Disagree
    - Strongly disagree

14) I think the COVID-19 vaccine will stop my adolescent child from passing the COVID-19 virus to others especially in our household.
    - Strongly agree
    - Agree
    - Neither agree or disagree
    - Disagree
    - Strongly disagree

Vaccinating under conditions of Uncertainty

15) From information that I have been able to find, I think the COVID-19 vaccine is safe for my adolescent child.
    - Strongly agree
    - Agree
    - I don't know
    - Disagree
    - Strongly disagree

Information sources

16) Indicate how much you trust vaccine information from medical professionals. For example, doctors.
    - Not at all
    - Slightly
    - Somewhat
    - Very much
    - Extremely

17) Indicate how much you trust vaccine information from medical journals.
    - Not at all
    - Slightly
    - Somewhat
    - Very much
    - Extremely

18) Indicate how much you trust vaccine information from newspaper articles.
    - Not at all
    - Slightly
    - Somewhat
    - Very much
    - Extremely

19) Indicate how much you trust vaccine information from new media websites. For example, local or national news.
    - Not at all
    - Slightly
    - Somewhat
    - Very much
    - Extremely

20) Indicate how much you trust vaccine information from social media. For examples, social networking sites and online blogs or vlogs.
    - Not at all
    - Slightly
    - Somewhat
    - Very much
    - Extremely

21) Indicate how much you trust vaccine information from family. For example, spouses or other relatives.
    - Not at all
    - Slightly
    - Somewhat

- Very much
- Extremely

22) Indicate how much you trust vaccine information from friends.

- Not at all
- Slightly
- Somewhat
- Very much
- Extremely

23) Indicate how much you trust vaccine information from public health websites. For example, the CDC, World Health Organization (WHO), and Orange County Department of Health

- Not at all
- Slightly
- Somewhat
- Very much
- Extremely

Ranking of vaccine concerns for parents and adolescents

24) When thinking about **vaccinating yourself**, please rank your top five COVID-19 vaccine **concerns**

1. Concerns over potential short term side effects or reactions (Example: pain)
2. Concerns over potential long term side effects
3. Concerns over the ingredients that make up the vaccine
4. Concerns over vaccine safety
5. Personal allergies
6. Personal medical history (Example: high risk health condition)
7. Fear of receiving too many vaccines
8. Concerns over how well the vaccine actually prevents COVID-19
9. New vaccine platform – mRNA vaccine
10. Trust in the scientists who make the vaccines
11. Trust in the vaccine regulatory agencies, such as the FDA
12. Trust in healthcare providers
13. Trust in government
14. Lack of support by important others (Example: family, friends, or political party)
15. Conspiracy theories
16. Low perceived risk of getting COVID-19
17. Low perceived risk of COVID-19 disease severity
18. Previously COVID-19 positive
19. Preference for natural immunity
20. Cost

21. Access
22. Fear of needles
23. Not enough vaccine information or a lack of access to vaccine information
24. Immigration status
25. No perceived need to vaccinate to protect vulnerable people
26. Information on social media
27. Traditional media sources (Example: TV or newspapers)
28. Uncertainty about vaccine benefits
29. Other (please indicate) ____

25) When thinking about vaccinating for **your adolescent child**, please rank your top five COVID-19 vaccine **concerns**

1. Concerns over potential short term side effects or reactions (Example: pain)
2. Concerns over potential long term side effects
3. Concerns over the ingredients that make up the vaccine
4. Concerns over vaccine safety
5. Concerns about child potentially having a strong allergic reaction to the COVID vaccine
6. Concern about child's medical history (Example: high risk health condition)
7. Fear of receiving too many vaccines
8. Concerns over how well the vaccine actually prevents COVID-19
9. New vaccine platform – mRNA vaccine
10. Trust in the scientists who make the vaccines
11. Trust in the vaccine regulatory agencies, such as the FDA
12. Trust in providers
13. Trust in government
14. Lack of support by important others (Example: other parent, family, friends, or political party)
15. Conspiracy theories
16. Low perceived risk of getting COVID-19
17. Low perceived risk of COVID-19 disease severity
18. Previously COVID-19 positive
19. Preference for natural immunity
20. Cost
21. Access
22. Fear of needles
23. Not enough vaccine information or a lack of access to vaccine information
24. Immigration status
25. No perceived need to vaccinate to protect vulnerable people
26. Information on social media
27. Traditional media sources (Example: TV or newspapers)
28. Uncertainty about vaccine benefits
29. Other (please indicate) ____

Ranking of vaccine motivators for parents and adolescents

26) When thinking about **vaccinating yourself**, please rank the top five things that would **motivate** you to get the COVID-19 vaccine

1. A healthcare provider recommendation
2. Confidence in vaccine safety
3. Confidence in how well the vaccine actually prevents COVID-19
4. Confidence in the vaccine development process
5. Trust in the scientists who make the vaccines
6. Trust in the vaccine regulatory agencies, such as FDA
7. Trust in healthcare providers
8. Trust in government
9. Support by important other (Example: family, friends, or political party)
10. Personal medical history (Example: High risk health condition)
11. Personal allergies
12. Lack of concern about short term side effects
13. Lack of concern about potential long term side effects
14. Lack of concern about the ingredients that make up the vaccine
15. High perceptions of risk of contracting COVID-19
16. High perceptions of COVID-19 disease severity
17. Having enough information or access to information
18. Not worried about cost
19. Not worried about access to get the vaccine
20. Information on social media
21. Traditional media sources (Example: TV or newspapers)
22. Certain about vaccine benefits
23. Social benefit (Example: return to normalcy)
24. A collective responsibility to protect others (Example: herd immunity)
25. Other (please indicate below) ____

27) When thinking about vaccinating for **your adolescent child**, please rank the top five things that would **motivate** you to get them the COVID-19 vaccine

1. A healthcare provider recommendation
2. Confidence in vaccine safety
3. Confidence in how well the vaccine actually prevents COVID-19
4. Confidence in the vaccine development process
5. Trust in the scientists who make the vaccines
6. Trust in the vaccine regulatory agencies, such as FDA
7. Trust in healthcare providers
8. Trust in government
9. Support by important other (Example: other parent, family, friends, or political party)
10. Child's medical history (Example: High risk health condition)
11. Child's allergies

12. Lack of concern about short term side effects
13. Lack of concern about potential long term side effects
14. Lack of concern about the ingredients that make up the vaccine
15. High perceptions of risk of contracting COVID-19
16. High perceptions of COVID-19 disease severity
17. Not worried about cost
18. Not worried about access to get the vaccine
19. Social benefit (Example: return to normalcy)
20. A collective responsibility to protect others (Example: herd immunity)
21. Having enough information or access to information
22. Information on social media
23. Traditional media sources (Example: TV or newspapers)
24. Certain about vaccine benefits
25. Other (please indicate below) ____

COVID vaccine history

28) Have you received the COVID-19 vaccine?
- Yes
- No

COVID vaccine likelihood

29) If you have not received the COVID-19 vaccine, on a scale from 1 to 10, how likely are you to get the COVID-19 vaccine when it becomes available to you? (1=not likely at all, 10=extremely likely). Please indicate your rating below. If not applicable, indicate N/A/

30) What is your age? _____

31) What is your gender?:
- Male
- Transgender, Male
- Female
- Transgender, Female
- Other (please specify) ____

32) Are you of Hispanic, Latino, or Spanish origin?
- Yes
- No

33) How would you describe yourself? (Please check all that apply)
- American Indian or Alaska Native

- Asian
- Black or African American
- Native Hawaiian or Other Pacific Islander
- White
- Other (please specify) ____

34) What is the highest level of education you have completed?
- Less than high school
- High school
- GED (General Education Diploma)
- Associate's degree (2-year college)
- Bachelor's degree
- Graduate school
- Some college
- Vocational/technical school
- Other (please specify) ____

35) Please indicate your occupation: ____

36) Were you born outside of the U.S.?
- Yes
- No

37) What is your political affiliation?
- Republican
- Democrat
- Independent
- Other (please specify) ____

38) How many members of your extended family reside in your household?: __________

39) Please list the family member's relation to you (Example: mother, grandmother, uncle)
____

40) How many child dependents do you have in your household? ___________________

41) What is the age of your adolescent child participating in the study? _________________________

42) What is your adolescent child's gender?:

- Male
- Transgender, male
- Female
- Transgender, female
- Other (please specify) ____

43) Is your adolescent child of Hispanic, Latino, or Spanish origin?
- Yes
- No

44) How would you describe your adolescent child? (Please check all that apply)
- American Indian or Alaska Native
- Asian
- Black or African American
- Native Hawaiian or Other Pacific Islander
- White
- Other (please specify) ____

45) What grade is your adolescent child in? _______________

46) Does your adolescent child have any chronic medical conditions? (Please check all that apply)
- Asthma
- Autism or the Asperger spectrum
- Autoimmune disease
- Cancer
- Cerebral palsy
- Cystic fibrosis
- Diabetes (Type 1 or Type 2)
- Obesity or overweight
- Mental illness
- Other (please specify) ____
- My child does not have any chronic medical conditions

47) In the past year, did your adolescent child receive the flu shot?
- Yes
- No
- I don’t remember

48) When your child started school, did they receive the following recommended vaccine for adolescents: Tdap vaccine? This vaccine protects against Tetanus, diphtheria, and acellular pertussis (whooping cough).

- Yes
- No
- I don't remember
- Other: ________________________________

49) When your child started school, did they receive the following recommended vaccine for adolescents: HPV vaccine? This vaccine can prevent infection with some types of human papillomavirus.

- Yes
- No
- I don't remember

50) When your child started school, did they receive the following recommended vaccine for adolescents: Meningococcal vaccine?

- Yes
- No
- I don't remember

51) Please indicate the type of school your adolescent child is currently enrolled in:

- Public
- Private
- Other: _______________________

52) As of today, does your adolescent child attend school in person?

- Yes
- No

53) During the pandemic, has your adolescent child been employed outside of the household as an essential worker? (Example: grocery store cashier, restaurant dishwasher, etc.)

- Yes
- No

54) Please indicate what type of work your adolescent child performed. Example: grocery or fast food worker. If not applicable, enter N/A ____
